# Ultrasensitive Near‐Infrared InAs Colloidal Quantum Dot‐ZnON Hybrid Phototransistor Based on a Gradated Band Structure

**DOI:** 10.1002/advs.202207526

**Published:** 2023-04-23

**Authors:** Jong‐Ho Kim, Byung Ku Jung, Su‐Kyung Kim, Kwang‐Ro Yun, Junhyuk Ahn, Seongkeun Oh, Min‐Gyu Jeon, Tae‐Ju Lee, Seongchan Kim, Nuri Oh, Soong Ju Oh, Tae‐Yeon Seong

**Affiliations:** ^1^ Department of Materials Science and Engineering Korea University 145 Anam‐ro, Seongbuk‐gu Seoul 02841 Republic of Korea; ^2^ Department of Nanophotonics Korea University 145 Anam‐ro, Seongbuk‐gu Seoul 02841 Republic of Korea; ^3^ Division of Materials Science and Engineering Hanyang University 222 Wangsimni‐ro, Seongdong‐gu Seoul 04673 Republic of Korea

**Keywords:** high mobility, hybrid phototransistor, low persistent photoconductivity effect, near‐infrared photodetection, non‐toxic materials

## Abstract

Amorphous metal oxide semiconductor phototransistors (MOTPs) integrated with colloidal quantum dots (QDs) (QD‐MOTPs) are promising infrared photodetectors owing to their high photoconductive gain, low off‐current level, and high compatibility with pixel circuits. However, to date, the poor mobility of conventional MOTPs, such as indium gallium zinc oxide (IGZO), and the toxicity of lead (Pb)‐based QDs, such as lead sulfide and lead selenide, has limited the commercial applications of QD‐MOTPs. Herein, an ultrasensitive QD‐MOTP fabricated by integrating a high‐mobility zinc oxynitride (ZnON)–based MOTP and lead‐free indium arsenide (InAs) QDs is demonstrated. A new gradated bandgap structure is introduced in the InAs QD layer that absorbs infrared light, which prevents carriers from moving backward and effectively reduces electron–hole recombination. Chemical, optical, and structural analyses confirm the movement of the photoexcited carriers in the graded band structure. The novel QD‐MOTP exhibits an outstanding performance with a responsivity of 1.15 × 10^5^ A W^−1^ and detectivity of 5.32 × 10^16^ Jones at a light power density of 2 µW cm^−2^ under illumination at 905 nm.

## Graphical abstract

A highly sensitive and non‐toxic near‐infrared–detecting phototransistor is fabricated by introducing a gradated band structure of indium arsenide quantum dots and combining it with zinc oxynitride. The responsivity, detectivity, and response time of phototransistors are characterized. The photocarrier behavior is analyzed to understand the mechanism for the enhanced performance of devices fabricated with a gradient gap.

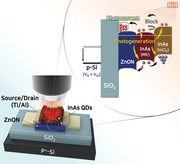

## 1 Introduction

The application of infrared (IR) photodetectors in autonomous vehicles, night vision systems, healthcare, and optical communications has attracted significant attention.^[^ ^1^, ^2^, ^3^ ^]^ Amorphous metal oxide semiconductor‐based thin‐film phototransistors (MOTPs) are being actively investigated as next‐generation photodetectors owing to their low off‐current level, high photoconductive gain, and high compatibility with pixel circuits. In particular, the carrier transporting layer with high mobility in MOTPs promotes the recirculation of the majority carriers between the source and drain until the minority carriers’ decay, thus increasing the photogain.^[^ ^1^ ^]^ However, MOTPs have shown limited performance due to the use of amorphous metal oxides with low mobility, such as indium gallium zinc oxide (IGZO) (<20 cm^2^ V^−1^ s^−1^); in addition, the wide optical bandgap of MOTPs limits the detection range of wavelengths (<450 nm), thus making it difficult to employ them in IR detection sensors.^[^ ^4^, ^5^ ^]^ Therefore, to develop MOTPs for high‐sensitivity IR detection, it is essential to adopt amorphous metal oxide semiconductors with high mobility and integrate the resulting MOTP with an IR‐absorbing material.

Compared to the typically used metal oxide semiconductors, zinc oxynitride (ZnON) has attracted significant attention owing to its high mobility (>50 cm^2^ V^−1^ s^−1^), low effective mass (0.19 m_e_), and small potential fluctuations in the conduction band minimum, which can improve the charge transportation between the source and drain electrodes, resulting in a high photogain.^[^ ^6^, ^7^, ^8^ ^]^ In addition, ZnON‐based MOTPs can minimize the persistent photoconductivity (PPC) effect, which, in turn, can increase the photoresponse time of MOTPs, because the N 2p orbital of ZnON covers the oxygen vacancy state (V_o_).^[^ ^9^, ^10^, ^11^ ^]^ Jang et al.^[^ ^10^ ^]^ reported that ZnON displayed a considerably higher mobility than IGZO (67.0 vs 8.42 cm^2^ V^−1^ s^−1^); in addition, the recovery time after turning off the light was significantly faster using ZnON, thereby indicating that the PPC effect was negligible for ZnON (unlike IGZO).

Colloidal quantum dots (CQDs) are promising IR light‐absorbing materials owing to their solution processability, high functionality, and large bandgap tunability.^[^ ^12^, ^13^, ^14^ ^]^ Especially, Pb‐based CQDs have been widely used in the fabrication of hybrid phototransistors comprising integrated CQDs and MOTPs (QD‐MOTPs). The excellent optoelectronic properties of CQDs, such as high absorption coefficient, low exciton binding energy, and high electron–hole mobility, imply that they can efficiently create excitons and transport photogenerated carriers to the MOTP, thereby resulting in high photoresponsivity.^[^ ^15^, ^16^, ^17^ ^]^ For example, Choi et al.^[^ ^18^ ^]^ achieved a responsivity of 10^4^ A W^−1^ and a detectivity of 10^11^–10^12^ Jones under 1310 nm laser illumination by integrating IGZO with lead sulfide (PbS) QDs for short‐wavelength IR detection. Furthermore, to obtain high photosensitivity under broad wavelengths (365–1310 nm), Kim et al.^[^ ^19^ ^]^ implemented a photodetector array circuit using IGZO and PbS QDs, and acquired a responsivity of over 8.3 × 10^3^ A W^−1^ and detectivity of 1.3 × 10^12^ Jones under 1310 nm illumination. However, despite the excellent photovoltaic properties of QD‐MOTPs, the use of Pb‐based QD devices for commercial applications is restricted because of their harmful effects on human health and the environment.^[^ ^20^ ^]^

Indium arsenide (InAs) CQDs have recently attracted great attention as lead‐free IR‐absorbing materials owing to their similar spectral range as PbS CQDs and outstanding optoelectrical properties stemming from high covalent such as low permittivity (*ε*_r_ = 6), low electron effective mass, and high chemical stability.^[^ ^21^, ^22^, ^23^, ^24^, ^25^ ^]^ Unfortunately, the physical properties of InAs CQDs are still not well understood. Therefore, the performance of InAs CQD‐based devices lags behind that of Pb CQD‐based devices. Furthermore, InAs CQDs require high surface energy to remove defects, which can decrease the responsivity of the QD‐MOTP and decrease their response speed, thus making it difficult to handle them and improve their quality.^[^ ^21^, ^26^, ^27^ ^]^ Consequently, most researchers investigating QD‐MOTPs still utilize Pb‐based QDs, while hitherto, the research on InAs QD‐based MOTPs has rarely been reported.

Herein, we report the fabrication of ultrasensitive InAs QD‐MOTPs using ZnON as a carrier transport layer, with the gradated bandgap structure of the InAs QD layer serving as an IR‐absorbing layer. To design the gradated bandgap structure, we utilized two different ligands, mercaptoethanol (ME) and indium chloride (InCl_3_), which affect the QD‐ligand surface dipole and shift energy diagram of InAs QDs, respectively.^[^ ^28^, ^29^ ^]^ Chemical, optical, and structural analyses were conducted, and the designed gradated conduction band level in each layer of the device was confirmed. Transient absorption spectral results show that the gradated band level effectively separates the electrons and holes in the QD layer and prolongs the recombination time of the excitons,^[^ ^30^ ^]^ thereby promoting carrier recycling in the ZnON layer. Consequently, the responsivity of the InAs QD‐MOTP was enhanced by 300% owing to the introduction of gradated band structure of the InAs QD layer, which is significantly better than that of other previously reported QD‐MOTPs.

## 2 Results and Discussion

To fabricate InAs QD‐MOTPs, InAs CQDs capped with long organic ligand molecules were synthesized according to the method reported previously^[^ ^22^ ^]^ with slight modifications and deposited on ZnON. To enhance the optoelectrical properties, long organic ligand molecules were exchanged with one of the two short ligands, 2‐mercaptoethanol (ME) or indium (III) chloride (InCl_3_). The ligand‐exchange process followed the procedure described in the Experimental section (Figure S1, Supporting Information). The chemical, optical, and structural properties of InAs CQD thin films are shown in Figure S2, Supporting Information. Figure S2a, Supporting Information, shows the UV–vis spectra of the InAs CQD thin films before and after surface ligand exchange. The spectrum of the pristine InAs CQD thin film showed a first exciton peak at 984 nm, which was red‐shifted to 1012 and 1010 nm in the spectra of ME‐ and InCl_3_‐treated InAs CQD thin films, respectively (hereafter referred to as ME‐InAs and InCl_3_‐InAs, respectively), owing to the reduced interparticle distance and increased coupling effect.^[^ ^31^ ^]^ Figure S2b, Supporting Information, shows the Fourier‐transform IR spectroscopy (FT‐IR) spectra of pristine InAs, InAs‐ME, and InAs‐InCl_3_ CQD thin films. The peaks at 2800–3000 and 1520 cm^–1^ correspond to the CH–stretching and carboxylate anions of long oleic acid (OA) molecules, respectively.^[^ ^32^, ^33^ ^]^ Compared to the FT‐IR spectrum of pristine InAs, these peaks were reduced by over 70% at the peaks of InAs‐ME and InAs‐InCl_3_, indicating that most of the original ligand had been eliminated. Additionally, in the FT‐IR spectrum of InAs‐ME, a peak appeared at 1042 cm^–1^, attributed to the bending vibration peak of the hydroxyl group in ME. Figure S2c, Supporting Information, exhibits the X‐ray photoelectron spectroscopy (XPS) of InAs CQDs films. After the ligand‐exchange process, the carbon peak originating from the original ligand dramatically decreased in intensity. Chloride and sulfur atoms were detected for InAs‐InCl_3_ and InAs‐ME, respectively, implying the occurrence of the exchange ligands on the InAs CQDs.

The structural properties of the InAs‐based CQDs were investigated via X‐ray diffraction (XRD) and transmission electron microscopy (TEM) (Figure S2d,e, Supporting Information, respectively). From the XRD and selected area electron diffraction patterns of InAs‐OA, the cubic zinc blend crystal structure of bulk InAs was confirmed. After the ligand‐exchange process, the XRD patterns of InAs‐ME and InAs‐InCl_3_ did not change, implying that the crystal structure remained after the ligand‐exchange process. The size of the pristine InAs CQDs was 4.2 ± 0.15 nm (Figure S2e, Supporting Information) with an interparticle distance of 8.05 ± 0.67 nm. The TEM images of InAs‐InCl_3_ and InAs‐ME revealed that the interparticle distance was dramatically decreased by exchanging the original ligand with ME or InCl_3_.

The grazing incidence X‐ray diffraction (GIXRD) patterns of the ZnON films before and after annealing are shown in Figure S3, Supporting Information. No evident diffraction peak was observed, indicating that the ZnON films had an amorphous structure, regardless of annealing.

To investigate the optoelectronic performance of two different types of MOTPs fabricated with ZnON and the conventional metal oxide semiconductor IGZO, these two materials were integrated with InAs‐ME. As described in Notes S1–S3, Supporting Information, ZnON was selected as the carrier transport layer in this study because the performance of ZnON phototransistors is superior to that of IGZO phototransistors (Figure S4a,b and Table S3, Supporting Information).

**Figure 1:**
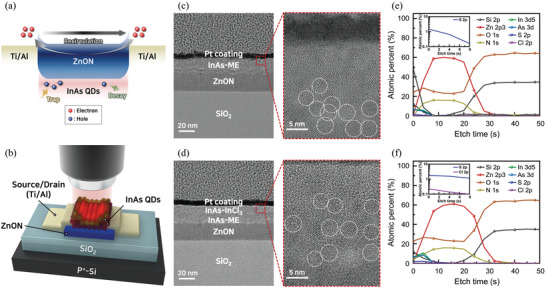
a) Schematic of photocurrent collection in ZnON/InAs QD phototransistors. b) Schematic of InAs QD‐MOTP. The cross‐sectional high‐resolution TEM images of the c) ZnON/InAs‐ME and d) ZnON/InAs‐ME/InAs‐InCl_3_ phototransistors, respectively. White dotted circles in magnified TEM images represent individual QDs. XPS depth profiles of the e) ZnON/InAs‐ME (inset: atomic percentage of S) and f) ZnON/InAs‐ME/InAs‐InCl_3_ (inset: atomic percentage of S and Cl) phototransistors, respectively.

Two different types of devices were prepared to examine the effects of the gradated bandgap structure. The ZnON/InAs‐ME device was fabricated by depositing InAs‐ME on ZnON, whereas the ZnON/InAs‐ME/InAs‐InCl_3_ device was fabricated by sequentially depositing InAs‐ME and InAs‐InCl_3_ on ZnON to form a gradated bandgap structure. **Figure** **1**a demonstrates the photocarrier behaviors in the ZnON/InAs phototransistors. The generated photocurrents (electrons) are repeatedly circulated and collected between the electrodes until the hole decays, which improves the photoresponses. Figure 1b illustrates the 3D configuration of the InAs QD‐MOTP. Figure 1c,d shows the cross‐sectional high‐resolution TEM images of the ZnON/InAs‐ME and ZnON/InAs‐ME/InAs‐InCl_3_ devices, respectively. In both cases, the thicknesses of the InAs QD layers were similar and ZnON was uniformly distributed.

XPS depth profiles were obtained to determine the junction formation of ZnON/InAs‐ME and ZnON/InAs‐ME/InAs‐InCl_3_. Figure 1e,f shows that the ZnON/InAs QD layers were stacked in order. However, the atomic percentages of S and Cl, the constituent elements of ME and InCl_3_, were significantly lower than those of other elements (Zn, O, N, In, and As). This is because the amounts of S and Cl in the ligand are small and each QD layer is very thin. To check the amounts of S and Cl more closely, the range was reduced to a low percentage (from 0.1% to 10%) and the initial part of the etching time (0 to 8 s), as shown in insets of Figure 1e. In the inset of Figure 1e, the distributions of Cl and S are shown; however, in the inset of Figure 1c, only the distribution of S is confirmed, implying that the InAs‐InCl_3_ layer was deposited on the InAs‐ME layer.

**Figure 2:**
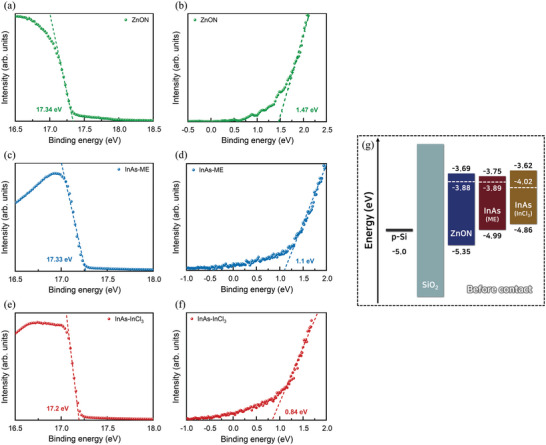
UPS spectra of the secondary cutoff regions of a) ZnON, c) InAs‐ME, and e) InAs‐InCl_3_ and of the onset regions of b) ZnON, d) InAs‐ME, and f) InAs‐InCl_3_. g) Band diagrams of ZnON, InAs‐ME, and InAs‐InCl_3_ before contact.

To reveal the charge transport mechanism, the band alignment between the ZnON and InAs QD layers was estimated using UV–vis spectroscopy and UV photoemission spectroscopy (UPS) measurements (**Figure** **2**). The positions of the Fermi levels and valence bands were calculated from the cutoff and onset regions in the UPS spectra. Figure 2a,c,e shows the secondary cutoff regions of ZnON, InAs‐ME, and InAs‐InCl_3_, respectively. The Fermi level (*E*_f_) was calculated using the following equation

(1) $$E_{f}=\mathrm{PE}-\left|E_{\mathrm{cut}-\mathrm{off}}\right|$$

where PE is the photon energy of the UV source (21.22 eV) and *E*_cut‐off_ is the estimated binding energy of the secondary cutoff.^[^ ^34^, ^35^ ^]^ The Fermi levels of ZnON, InAs‐ME, and InAs‐InCl_3_ were positioned at −3.88, −3.89, and −4.02 eV, respectively. In addition, the difference between the work function and valence‐band edge values was determined from the onset regions of the UPS spectra. As shown in Figure 2d,f the valence‐band maxima of ZnON, InAs‐ME, and InAs‐InCl_3_ were −5.35, −4.99, and −4.86 eV, respectively. The Tauc plots of ZnON before and after annealing (Figure S5a, Supporting Information) were obtained from the UV–vis absorbance spectra and the relationship between the absorption coefficient (*α*) and bandgap (*E*_g_) as follows:

(2) $$\alpha h\nu \propto {\left(h\nu -E_{g}\right)}^{n}$$

where *hν* is the photon energy. Since ZnON has an indirect energy bandgap, *n* = 2 in Equation (2).^[^ ^36^, ^37^ ^]^ Moreover, ZnON exhibited a bandgap of 1.66 eV, which is slightly wider than previously reported values,^[^ ^38^, ^39^ ^]^ which resulted from the annealing process changing the oxygen and nitrogen concentrations in the ZnON layer (Figure S5b,c and Note S4, Supporting Information). The bandgaps of InAs‐ME and InAs‐InCl_3_ were calculated from the first exciton energy in the UV–vis spectra (Figure S2a, Supporting Information), and the total energy band diagrams are shown in Figure 2g. The electrical properties of the two films were assessed using space‐charge–limited current measurement, as detailed in Figure S6, Supporting Information.

**Figure 3:**
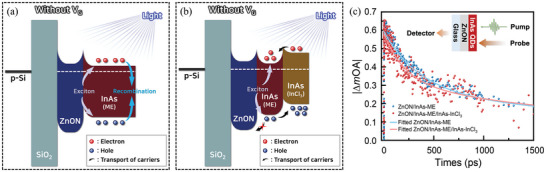
Exciton diffusion schematic of a) ZnON/InAs‐ME and b) ZnON/InAs‐ME/InAs‐InCl_3_ under no gate bias applied. c) Transient absorption decay kinetics of ZnON/InAs‐ME and ZnON/InAs‐ME/InAs‐InCl_3_ tracked at the first exciton peak of InAs CQDs (1020 nm) under excitation with pump beam at 450 nm. Inset briefly illustrates the TA measurement process of samples (Glass/ZnON/InAs QDs).

To investigate the photo‐induced carrier behavior, time‐resolved transient absorption spectroscopy (Tr‐TAS) was conducted under the no gate bias condition (**Figure** **3**a,b): Δ*OA* = *OA*_1_ − *OA*_0_, where *OA*_0_ and *OA*_1_ are the absorbances of the samples at 1000 nm before and after exposure to 450 nm pump light, respectively, detailed in the Experimental Section. The absorbance signals of both ZnON/InAs‐ME and ZnON/InAs‐ME/InAs‐InCl_3_ rapidly decreased after exposure and then gradually recovered (Figure 3c). Quantitative analysis of the Tr‐TA spectra was performed using the double exponential decay equation

(3) $$\left|\Delta OA\left(\tau \right)\right|=\sum_{i=1}^{2}A_{i}\left[1-e^{-\frac{t}{\tau_{i}}}\right]$$

where *∆OA(τ)* is the transient absorption signal, *A_i_* is the amplitude of the decay kinetics, and *τ_i_* is the decay time constant (Table S1, Supporting Information).^[^ ^40^ ^]^ The short‐time constant (*τ*_1_) and long‐time constant (*τ*_2_) are related to the interfacial carrier transfer and charge recombination processes occurring in the absorption layer. The *τ*_2_ value of ZnON/InAs‐ME/InAs‐InCl_3_ (1683.69 ps) was slower than that of ZnON/InAs‐ME (1198.62 ps); this longer *τ*_2_ implies an increased recombination lifetime of the remaining photogenerated charge carriers in the InAs layer, in line with the expected charge separation by the built‐in potential of the graded bandgap structure (Figure 3a,b).^[^ ^1^, ^24^, ^41^, ^42^, ^43^, ^44^, ^45^, ^46^ ^]^

**Figure 4:**
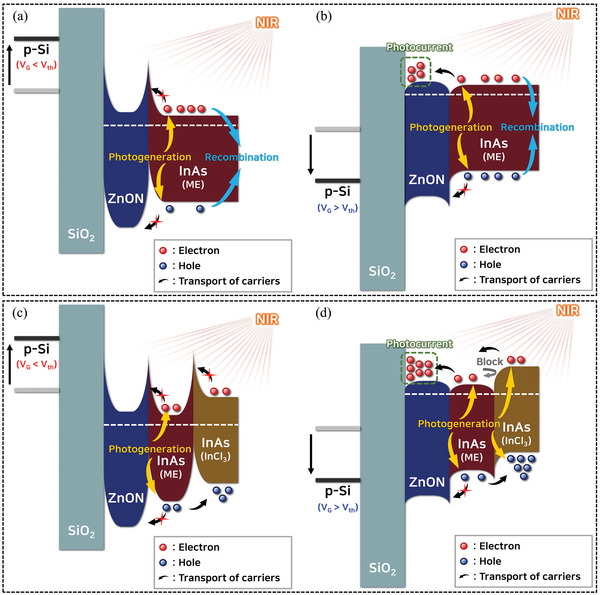
Exciton diffusion schematic of a,b) ZnON/InAs‐ME and c,d) ZnON/InAs‐ME/InAs‐InCl_3_ depending on the gate bias.

Based on the Tr‐TAS results, the carrier diffusion schematics of ZnON/InAs‐ME and ZnON/InAs‐ME/InAs‐InCl_3_ under highly negative and positive gate bias conditions are presented in **Figure** **4**a–d. Figure 4a–d shows the energy barriers at each interface (ZnON/InAs‐ME and InAs‐ME/InAs‐InCl_3_). The barriers existing in the conduction band at each interface result from the difference in the work function between layers, and it is assumed that the equilibrium of the overall Fermi levels is formed at the average Fermi level of each layer. The heights of these barriers depend on the change in the bias. When the gate voltage is lower than the threshold voltage (off state), the Fermi level of each layer moves downward in the opposite direction of the gate bias. Conversely, when the gate voltage is higher than the threshold voltage (on state), the Fermi level of each layer moves upward in the opposite direction to the gate bias, thus lowering the height of the barrier. Similarly, in the on‐state valence band, barriers are formed owing to the Fermi level shift. Figure 4a,b illustrates the operating mechanism of ZnON/InAs‐ME in the off and on states under 905 nm laser illumination. In the off state (Figure 4a), it is difficult to transfer electrons generated from InAs‐ME to the ZnON layer because of the energy barrier caused by the high negative gate bias and the difference in work function between ZnON and InAs‐ME in the conduction band. In the on state (Figure 4b), photogenerated holes and electrons behave differently. While transferring the holes to the ZnON layer is difficult owing to the valence‐band offset (Note S5, Supporting Information) and barriers, the electrons can be readily transferred to the ZnON layer. Trapped holes remain until electron–hole recombination occurs in the QD layer, and electron recycling is induced in the ZnON layer. Figure 4c,d shows the operating mechanism of the ZnON/InAs‐ME/InAs‐InCl_3_ device. In the off state (Figure 4c), electron transportation from the QD layer to the ZnON layer is also difficult owing to the negative gate bias (*EB*_ZnON/ME_) and the additional energy barrier between the InAs‐ME and InAs‐InCl_3_ layers. In the on state (Figure 4d), the InAs‐ME and InAs‐InCl_3_ layers form a gradated conduction band level. The InAs‐InCl_3_ layer partially separated the photogenerated holes and electrons, which suppressed the electron–hole recombination process and accelerated the electron recycling process more in the ZnON layer than in the ZnON/InAs‐ME device.

**Figure 5:**
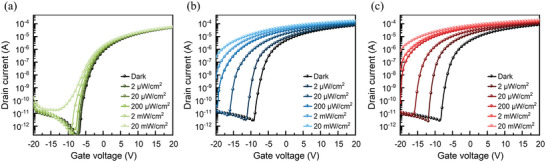
Transfer characteristics of a) ZnON‐only, b) ZnON/InAs‐ME, and c) ZnON/InAs‐ME/InAs‐InCl_3_ devices under no illumination and 905 nm laser illumination intensities of 2 µW cm^−2^, 20 µW cm^−2^, 200 µW cm^−2^, 2 mW cm^−2^, and 20 mW cm^−2^.

**Figure** **5**a–c presents the transfer characteristics of the ZnON‐only, ZnON/InAs‐ME, and ZnON/InAs‐ME/InAs‐InCl_3_ devices. To obtain data for the transfer curves, the drain current was measured while sweeping the gate voltage from −20 to 20 V at a drain voltage of 10 V. The saturation mobility (*µ*_Sat_), *V*_th_, subthreshold swing (SS), and on/off current ratio (*I*_on/off_) of each device in the dark state are listed in Table S2, Supporting Information. The ZnON‐only device exhibited a *µ*_Sat_ value of 73.8 cm^2^ V^−1^ s^−1^, thereby inferring excellent carrier transport characteristics and its applicability as a carrier transport layer. Although there is an additional InAs QD layer deposited on the ZnON layer in the ZnON/InAs‐ME and ZnON/InAs‐ME/InAs‐InCl_3_ devices, low SS values of around 0.2 V dec^−1^ were maintained in the dark state, similar to that of the ZnON‐only device. In addition, under a high negative bias below −15 V, there was no significant difference between the dark current values of the ZnON‐only, ZnON/InAs‐ME, and ZnON/InAs‐ME/InAs‐InCl_3_ devices because of the high negative gate bias and energy barrier blocking carrier transportation from the QD layer to the ZnON layer. Different behaviors were observed under low negative and positive biases (above −10 V). The *µ*_Sat_ values of the ZnON‐only, ZnON/InAs‐ME, and ZnON/InAs‐ME/InAs‐InCl_3_ devices were 73.8, 108.3, and 120.1 cm^2^ V^−1^ s^−1^, respectively, while their *I*_on/off_ values were 1.7 × 10^6^, 5.9 × 10^6^, 7.4 × 10^6^, respectively. These behaviors are attributed to the dark carriers thermally generated in the InAs layer filling up trap states (localized tail states) near the conduction band of the ZnON layer. Furthermore, the built‐in potential generated by the gradient bandgap causes more dark carriers to transfer to the ZnON and fill the trap states.

To measure the photoresponse characteristics of the devices, gate voltage versus source–drain current curves were generated by introducing light at 905 nm with varying power densities (from 2 µW cm^−2^ to 20 mW cm^−2^). As the light intensity increased, the transfer curves shifted toward the negative voltage side. For the ZnON‐only phototransistor, slight changes in the threshold voltage (Δ*V*_th_) of −0.2, −0.4, −0.9, −1.8, and −3.6 V occurred at the light intensities of 2 µW cm^−2^, 20 µW cm^−2^, 200 µW cm^−2^, 2 mW cm^−2^, and 20 mW cm^−2^, respectively (Figure 5a). This indicates that ZnON has a slight photoresponse to detect a near‐IR (NIR) wavelength of 905 nm. This is attributed to the presence of nitrogen‐related defect states, which are discussed in Note S4, Supporting Information. At light intensities of 2, 20, and 200 µW cm^−2^, the ZnON/InAs‐ME device incurred large threshold voltage shifts of −1.9, −6.8, and −11.2 V, respectively (Figure 5b), while the ZnON/InAs‐ME/InAs‐InCl_3_ device incurred even larger threshold voltage shifts of −3.5, −8.2, and −11.7, respectively (Figure 5c), compared to the ZnON‐only device. The *V*_th_ shift values of both the ZnON/InAs‐ME and ZnON/InAs‐ME/InAs‐InCl_3_ phototransistors could not be clearly measured above a light intensity of 2 mW cm^−2^ because numerous photons generated a high photo‐induced current exceeding the *V*_th_ reference point.

The significant difference in the *V*_th_ shift between the devices with and without the InAs QD layer indicates that the InAs QDs effectively absorbed NIR light and induced electron recycling in the ZnON layer (Figure S7, Supporting Information). In particular, the ZnON/InAs‐ME/InAs‐InCl_3_ phototransistor exhibited a larger *V*_th_ shift than the ZnON/InAs‐ME phototransistor, indicating that the InAs‐InCl_3_ layer effectively separated the photogenerated electrons and holes, and enhanced electron recycling in the ZnON layer (Figure 4).

To quantify the photoresponse performance of the ZnON/InAs‐ME and ZnON/InAs‐ME/InAs‐InCl_3_ phototransistors, the responsivity (*R*), detectivity (D*), linear dynamic range (LDR), photosensitivity (*S*), and external quantum efficiency (EQE) were calculated from Figure 5b,c using the following equations

(4) $$I_{\mathrm{ph}}=I_{\mathrm{light}}-I_{\mathrm{dark}}$$

(5) $$R=\frac{I_{\mathrm{ph}}}{PA}$$

(6) $$D^{*}=\frac{RA^{\frac{1}{2}}}{{\left(2qI_{\mathrm{dark}}\right)}^{\frac{1}{2}}}$$

(7) $$\mathrm{LDR}=20\log\left(\frac{P_{\max}}{P_{\min}}\right)$$

(8) $$S=\frac{I_{\mathrm{ph}}}{I_{\mathrm{dark}}}$$

(9) $$\mathrm{EQE}=\frac{Rhc}{q\lambda }\times 100\left(\%\right)$$

where *I*_light_ and *I*_dark_ are the current values in the illuminated and dark states, respectively; *P* is the light power density in the illuminated area; *A* is the area exposed to light; *q* is the electron charge; *h* is the Planck constant; *c* is the velocity of light; *λ* is the wavelength of the light; and *P*_max_ and *P*_m_ *_i_*_n_ are the maximum and minimum light power densities of the linear region of the photocurrent, respectively.^[^ ^1^, ^47^, ^48^ ^]^

**Figure 6:**
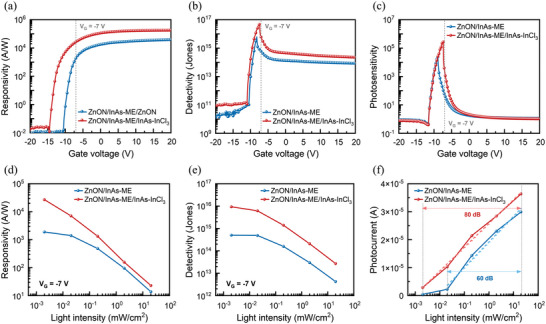
Gate voltage versus a) responsivity, b) detectivity, and c) photosensitivity plots for the ZnON/InAs‐ME and ZnON/InAs‐ME/InAs‐InCl_3_ devices under 905 nm illumination at 2 µW cm^−2^. Light intensity versus d) responsivity, e) detectivity, and f) photocurrent plots under different optical light power densities at a gate voltage of −7 V.

The responsivity variance as a function of gate voltage at the weakest light intensity of 2 µW cm^−2^ is shown in **Figure** **6**a. The ZnON/InAs‐ME/InAs‐InCl_3_ phototransistor attained a higher value than the ZnON/InAs‐ME phototransistor across the entire voltage range and showed a maximum value of 1.15 × 10^5^ A W^−1^ at a gate voltage of 20 V, which is threefold higher than that of the ZnON/InAs‐ME phototransistor (Figure S8a,d, Supporting Information). The dependence of the detectivity on the gate voltage at 2 µW cm^−2^ is illustrated in Figure 6b. The highest detectivity of 5.32 × 10^16^ Jones was observed for the ZnON/InAs‐ME/InAs‐InCl_3_ phototransistor, while that of the ZnON/InAs‐ME was 6.09 × 10^15^ Jones. Figure 6c shows the dependence of photosensitivity on the gate voltage at 2 µW cm^−2^. Although the photosensitivity values of the two devices started to increase at almost the same gate voltage, a rapid decrease in photosensitivity was detected at −9 and −7.7 V for the ZnON/InAs‐ME and ZnON/InAs‐ME/InAs‐InCl_3_ devices, respectively. Since the two devices showed similar off‐currents, this difference can be ascribed to the improved recirculation effect, resulting in an enhanced *V*_th_ shift due to the gradated bandgap structure (Figure 4b,d).

To investigate the dependence of the two devices on the light power density, responsivity, detectivity, and photocurrent were calculated at various intensities of the 905 nm light and the same gate voltage of −7 V. The responsivity values of the ZnON/InAs‐ME and ZnON/InAs‐ME/InAs‐InCl_3_ phototransistors are shown in Figure 6d and are listed in **Table** **1**. The ZnON/InAs‐ME/InAs‐InCl_3_ phototransistor shows higher responsivity across the entire intensity range than ZnON/InAs‐ME. However, the differences in responsivity decreased with the increasing light intensity, implying that more recombination between electrons and holes occurred in both devices due to the increased number of photoexcited carriers.^[^ ^49^, ^50^, ^51^ ^]^ Figure 6e illustrates the detectivity values of the ZnON/InAs‐ME/InAs‐InCl_3_ and ZnON/InAs‐ME phototransistors estimated at the optimized gate voltage of −7 V as a function of illumination power, which are also listed in Table 1. As the light intensity increased, the detectivity values of the ZnON/InAs‐ME decreased from 5.02 × 10^14^ to 4.24 × 10^12^ Jones while those of the ZnON/InAs‐ME/InAs‐InCl_3_ decreased from 9.38 × 10^15^ to 2.72 × 10^13^ Jones. As shown in Figure S8b,e, Supporting Information, the highest detectivity was obtained at the weakest light power density, owing to the highest responsivity. Figure 6f shows the LDR values, which are indicators of the range of illuminated light over the linear region of the photoresponse at the optimized gate voltage of −7 V. It is evident that the ZnON/InAs‐ME/InAs‐InCl_3_ phototransistor exhibited almost linear photocurrent characteristics in the range of 2 µW cm^−2^ to 20 mW cm^−2^ whereas that of the ZnON/InAs‐ME device sharply increased in the range of 20 to 200 µW cm^−2^. Using Equation (7), the LDR values were calculated as 80 and 60 dB for the ZnON/InAs‐ME/InAs‐InCl_3_ and ZnON/InAs‐ME devices, respectively. The wider LDR of the ZnON/InAs‐ME/InAs‐InCl_3_ phototransistor indicated a significant improvement in photodetection under weak light, indicating that subtle contrasts in an image can be better distinguished.^[^ ^1^, ^52^, ^53^, ^54^ ^]^ Figure S9a, Supporting Information, and Table 1 display the dependence of photosensitivity on the light power density at the optimized gate voltage of −7 V. In fact, the difference in photosensitivity between the two devices was maintained within a factor of 10^2^–10^3^ regardless of the light intensity. At a gate voltage of −7 V, the ZnON/InAs‐ME/InAs‐InCl_3_ phototransistor exhibited higher photocurrent and lower dark current values owing to the gradated bandgap structure, resulting in a higher photosensitivity value than the other devices. Figure S8c,f, Supporting Information, illustrates the photosensitivity of the two samples as a function of the gate voltage and light intensity. Figure S9b and Table S4, Supporting Information, show the EQE of the two devices depending on the light power density. The EQE was increased about ten times when using the gradient band structure (at *V*_G_ of −7 V and light power density of 2 µW cm^−2^). The maximum EQE (at *V*_G_ of 20 V and light power density of 2 µW cm^−2^) of the ZnON/InAs‐ME/InAs‐InCl_3_ phototransistor was 1.58 × 10^7^%, indicating a value greater than 100%, which is caused by the process described below (Figure 1a): First, the photo‐excited electrons and holes generated by light exposure prefer to migrate to ZnON and InAs‐InCl_3_ by the built‐in potential, respectively. At this time, holes are trapped at the trap sites of InAs‐InCl_3_, whereas electrons move to ZnON and circulate between the electrodes until holes (trapped in the InAs‐InCl_3_ layer) decay. Therefore, in ZnON, the faster the electron circulation rate between the electrodes (meaning the higher mobility of the ZnON) and the longer the hole decay time, the more electrons recirculate between the electrodes, increasing the photogain. Further, this phenomenon can cause an EQE of 100% or higher because one hole creates multiple electrons. In addition, we present the performance of ZnON/InAs‐InCl_3_ and compare it with the two devices in Figure S10a,b, Supporting Information.

**Table 1:** Calculated responsivity, detectivity, and photosensitivity values of ZnON/InAs‐ME and ZnON/InAs‐ME/InAs‐InCl_3_ under various light power densities at a gate voltage of −7 V

Light power density [W cm^−2^]	Responsivity [A W^−1^]		Detectivity [Jones]		Photosensitivity	
	ZnON/InAs‐ME	ZnON/InAs‐ME/InAs‐InCl_3_	ZnON/InAs‐ME	ZnON/InAs‐ME/InAs‐InCl_3_	ZnON/InAs‐ME	ZnON/InAs‐ME/InAs‐InCl_3_
2 × 10^−6^	1.88 × 10^3^	2.71 × 10^4^	5.02 × 10^14^	9.38 × 10^15^	3.81 × 10^2^	1.16 × 10^5^
2 × 10^−5^	1.40 × 10^3^	7.11 × 10^3^	4.91 × 10^14^	6.18 × 10^15^	4.83 × 10^3^	1.48 × 10^6^
2 × 10^−4^	4.80 × 10^2^	1.33 × 10^3^	1.58 × 10^14^	1.20 × 10^15^	1.53 × 10^4^	4.67 × 10^6^
2 × 10^−3^	9.50 × 10^1^	1.56 × 10^2^	2.98 × 10^13^	2.09 × 10^14^	2.65 × 10^4^	8.09 × 10^6^
2 × 10^−2^	1.37 × 10^1^	2.20 × 10^1^	4.24 × 10^12^	2.72 × 10^13^	3.48 × 10^4^	1.06 × 10^7^

Figure S11a,b, Supporting Information, shows the transient photoresponses of ZnON/InAs‐ME and ZnON/InAs‐ME/InAs‐InCl_3_ phototransistors. The rise time of the ZnON/InAs‐ME/InAs‐InCl_3_ device (2.43 s) is similar to that of the ZnON/InAs‐ME device (2.90 s). Meanwhile, the fall time (related to the storage of minority carriers in the light‐absorbing layer)^[^ ^55^, ^56^ ^]^ was significantly longer in ZnON/InAs‐ME/InAs‐InCl_3_ (4.16 s) than in ZnON/InAs‐ME (3.44 s). These results are attributed to the gradated bandgap structure, which prolongs the hole carrier lifetime in the QD layer and thereby induces longer electron recycling and fall times.

To examine the photoresponse that depends on the wavelength of the light source, performance at 980 nm was also investigated. Figure S12a,b, Supporting Information, shows responsivity and detectivity at a gate voltage of −7 V and light power density of 2 mW cm^−2^, respectively. The responsivity of ZnON/InAs‐ME is 2.10 × 10^1^ A W^−1^ at 980 nm, which is lower than that at 905 nm (9.52 × 10^1^ A W^−1^). Similarly, ZnON/InAs‐ME/InAs‐InCl_3_ gives responsivity of 5.47 × 10^1^ A W^−1^, which is lower than that at 905 nm (1.56 × 10^2^A W^−1^). The detectivity of ZnON/InAs‐ME is 4.5 × 10^13^ Jones at 980 nm, which is higher than that at 905 nm (2.98 × 10^13^ Jones), while that of ZnON/InAs‐ME/InAs‐InCl_3_ is 6.5 × 10^13^ Jones, which is lower than that at 905 nm (2.09 × 10^14^ Jones).

We examined the stability by comparing the devices before and after aging for a week in air. Figure S13a,b, Supporting Information, shows the changes in the transfer characteristics of the ZnON/InAs phototransistors under illumination. In the illuminated state, the devices were exposed to a 905 nm laser light of 2 mW cm^−2^. The overall current level decreased when aged in air for a week, compared to the immediately fabricated device. However, a reversal of the dark current is observed at gate voltages higher than 5.5 V (Figure S13a, Supporting Information), which is due to the deviation of the devices. Meanwhile, the ZnON/InAs‐ME/InAs‐InCl_3_ phototransistors exhibited an overall decrease in current (Figure S13b, Supporting Information). The photocurrent (*I*_Ph_) level changes of the ZnON/InAs QD phototransistors are illustrated in Figure S13c, Supporting Information. CR of *I*_Ph_ was calculated using the following equation

(10) $$\mathrm{CR}\left(I_{\mathrm{Ph}}\right)=\frac{\left(I_{\mathrm{Ph},\;\mathrm{After}\;1\;\mathrm{week}}-I_{\mathrm{Ph},\;\mathrm{As}-\mathrm{fabricated}}\right)}{I_{\mathrm{Ph},\;\mathrm{As}-\mathrm{fabricated}}}\times 100\left(\%\right)$$

where *I*_Ph, As‐fabr_ *_i_*_cated_ is the measured current immediately after fabrication and *I*_Ph, After 1 week_ is the measured current after aging for a week in air. As presented in Figure S13a,b, Supporting Information, the *I*_Ph_ of ZnON/InAs‐ME/InAs‐InCl_3_ phototransistor was reduced by at least 18.4% (Figure S13c, Supporting Information). Meanwhile, the *CR* of *I*_Ph_ shows a peak in the ZnON/InAs‐ME phototransistor due to the increase of *I*_Dark_ and the decrease of *I*_Light_ at the gate voltages higher than 5.5 V. Figure S13d,e, Supporting Information, displays changes in the maximum responsivity of the ZnON/InAs‐ME and ZnON/InAs‐ME/InAs‐InCl_3_ phototransistors before and after exposure to air. The maximum responsivity was calculated at a light power density of 2 mW cm^−2^, and the values of as‐fabricated devices were set to 100%. As shown in Figure S13d, Supporting Information, the maximum responsivity of ZnON/InAs‐ME phototransistors decreased by 28.2% after aging for a week in air. Meanwhile, the maximum responsivity of ZnON/InAs‐ME/InAs‐InCl_3_ phototransistors decreased by 20.1% (Figure S13e, Supporting Information). A decrease in the photocurrent, as illustrated in Figure S13c, Supporting Information, was the reason for the reduction in the maximum responsivity.

**Figure 7:**
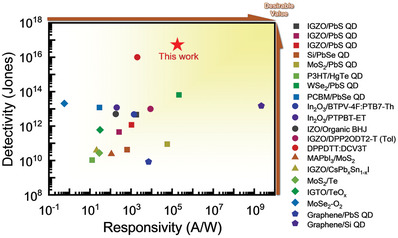
Comparison of the performances of the InAs QD‐MOTP developed in this study and previously reported NIR phototransistors.

The responsivity and detectivity values of previously reported NIR photodetectors fabricated with various materials and structures are shown in **Figure** **7** and summarized in Table S5, Supporting Information. Although Si QD/graphene devices exhibit ultrahigh responsivity, they display relatively low detectivity because of the high conductivity and dark current of graphene, thereby resulting in poor compatibility with pixel circuits. The performance of the ZnON/InAs‐ME/InAs‐InCl_3_ phototransistor was comparable to or superior to that of other NIR detection devices fabricated with various absorption materials. In particular, our device outperformed most Pb‐based devices, demonstrating the possibility of using eco‐friendly light absorbers in the fabrication of photodetectors. Moreover, our ZnON/InAs phototransistors with a gradated bandgap structure achieved high responsivity in the NIR region with excellent detectivity.

## 3 Conclusions

We report, for the first time, the fabrication of novel phototransistors consisting of InAs QDs and ZnON exhibiting outstanding performance. A superior photoresponse was achieved by combining the benefits of high NIR absorbance by the InAs QDs and rapid charge transfer to the electrode by ZnON. Moreover, we developed InAs CQDs thin film–based graded layers for the first time. These well‐aligned band structures of InAs, along with the improved recirculation effect of ZnON, enhanced the spatial separation of the photogenerated electrons and holes. Owing to this combination of excellent light‐absorbing properties and mobility, the ZnON/InAs‐ME/InAs‐InCl_3_ phototransistor exhibited a responsivity of over 10^5^ A W^−1^ and a detectivity of over 10^16^ Jones. These results indicate that ZnON/InAs hybrid phototransistors with a graded bandgap structure offer a highly promising approach for fabricating next‐generation photodetectors for NIR applications.

## 4 Experimental Section

### Materials

Indium acetate (InOAc, 99.99%), OA (≥99%), dioctylamine (DOA, ≥97%), 1‐octadecene (ODE, 90%), ME (≥99%), and InCl_3_ (≥98%) were purchased from Sigma‐Aldrich Chemical Co. Trimethylsilylarsine ((TMSi)_3_As, 99%) was purchased from JSI Silicone. All solvents used in the experiments (hexane, butanol, and ethyl alcohol) were purchased from Sigma‐Aldrich Chemical Co.

### The Synthesis of Colloidal InAs CQDs

These were synthesized via a continuous injection synthesis method previously reported in^[^ ^22^ ^]^ with slight modifications. An In seed solution was prepared using 0.29 g of InOAc, 0.85 g of OA, and 5 mL ODE and degassed in a 100 mL flask for 2 h in vacuum. The solution was then heated to 300 °C under an inert gas. An As seed solution comprising 0.14 g of (TMSi)_3_As, 0.36 g of DOA, and 1 mL of ODE was mixed and kept at 60 °C for 1 h in a glove box. When the As solution turned brown, it was rapidly injected into the prepared In seed solution at 300 °C. The temperature was maintained at 287 °C for 20 min.

The cluster solution comprising 1.74 g of InOAc, 5.10 g of OA, and 30 mL of ODE was degassed in a 100 mL flask for 2 h under vacuum. After degassing, the cluster solution was cooled to 30 °C under inert gas atmosphere. An As seed solution comprising 0.84 g of (TMSi)_3_As, 2.17 g of DOA, and 6 mL of ODE was mixed and kept at 60 °C for 1 h in a glove box. It was then rapidly injected into the prepared In seed solution under constant stirring at room temperature for 10 min.

The cluster solution was loaded into a syringe (diameter: 22 mm) and injected at 0.5 mL min^−1^ into the InAs CQD seed solution at 300 °C. Subsequently, the solution was cooled to room temperature, and the purification process was conducted in a glove box. The synthesized InAs CQD solution was divided into 10 mL aliquots. The anti‐solvent butanol (40 mL) was added to each sample, followed by centrifugation at 6000 rpm for 5 min. The precipitate was then dissolved in hexane (10 mL). To eliminate the by‐product indium oxide (In_2_O_3_), 15 mL butanol was added, followed by centrifugation at 4000 rpm for 5 min. The supernatant was collected and 20 mL of butanol was added, followed by centrifugation at 6000 rpm for 5 min. The precipitate was dissolved in 10 mL hexane, after which 30 mL butanol was added, followed by centrifugation at 6000 rpm for 5 min. These steps were repeated twice. Finally, the precipitate was dried under vacuum for 2 h before being dispersed in octane (10 mg mL^−1^).

### Fabrication of the Hybrid Phototransistors

Bottom‐gate ZnON/InAs QD phototransistors were fabricated on p^++^‐Si/silicon dioxide (SiO_2_) (300 nm) substrates (p^++^‐Si and SiO_2_ were used as the gate and gate insulator of the devices, respectively). The p^++^‐Si/SiO_2_ substrates were cleaned with acetone, isopropyl alcohol, and deionized water in an ultrasonic bath, and then dried under a nitrogen stream. An RF magnetic sputtering system was used to deposit 30 nm‐thick ZnON films on the p^++^‐Si/SiO_2_ substrates using a Zn target (99.99% purity). The sputtering power, working pressure, and oxygen/nitrogen flow rate were 100 W, 5 mTorr, and 0.02, respectively. For the source/drain electrodes, a Ti (5 nm)/Al (100 nm) layer patterned using a shadow mask was deposited via e‐beam evaporation. Subsequently, post‐annealing was conducted at 250 °C for 1 h in air. The width and length of the channel were 50 and 200 µm, respectively. For the InAs‐ME/IGZO phototransistor, an IGZO film was deposited via RF sputtering in Ar ambient with a sputtering power and working pressure of 100 W and 5 mTorr, respectively, after which source/drain electrodes (Ti/Al) were deposited using an e‐beam evaporator. Post‐annealing was performed at 400°C for 1 h in air. The thicknesses of the layers in the IGZO phototransistors were the same as those in the ZnON phototransistors.

To fabricate the ZnON/InAs‐ME device, as‐synthesized OA‐capped InAs QDs (10 mg mL^−1^ in octane) were spin‐coated onto a ZnON thin film at a rotation speed of 5000 rpm for 30 s. Next, to exchange the OA ligand with ME, the films were covered with an ME solution (5 mm in ethanol) for 30 s and then washed with ethanol three times. This washing process was conducted only to remove the residual ligands, and there was no ligand loss during washing (Figure S1, Supporting Information). These steps were repeated. To fabricate the ZnON/InAs‐ME/InAs‐InCl_3_ device, a graded bandgap structure was formed by depositing InAs QD and InAs‐InCl_3_ layers on top of the InAs‐ME layer. The as‐synthesized QDs were spin‐coated onto the InAs‐ME layer, and the structure was then covered with an InCl_3_ solution (5 mm in ethanol) for 30 s. The film was then washed three times with ethanol.

### Characterization

The performance of the devices was measured using an Agilent 4155C and laser diodes (905 and 980 nm) as the light source. The threshold voltage was defined as the voltage at a current of 2.5 × 10^−10^ A (1 nA × channel width/channel length). The depth profile of ZnON was investigated using Auger electron spectroscopy with Ar^+^ sputtering (PHI‐710, ULVAC‐PHI). The GIXRD spectra were obtained using (Dmax2500/PC, Rigaku). XPS depth profiling was conducted to confirm the elemental distribution along the junction (PHI 5000 VersaProbe, ULVAC‐PHI). A UV–vis spectrometer (UV‐1800, Shimadzu) was used to calculate the optical bandgap of ZnON. UPS (AXIS NOVA, Kratos Analytical) was used to obtain the spectra of the ZnON and QD layers. The band structures of the InAs QDs and ZnON were measured by combining the UPS results with the optical band gaps. The thicknesses of the multilayered devices were measured using Cs‐TEM (JEM‐ARM 200F, JEOL Ltd.), and the elemental distributions in the ZnON and InAs QD layers were visualized through EDS mapping. FT‐IR (iS50, Nicolet) samples were prepared as follows: InAs CQDs were spin‐coated as thin films on amorphous SiO_2_ slide glass to analyze the attenuated total reflectance. From SCLC curve, the trap density (*N*_trap_) and electron mobility (µ) of two films were determined by the following equations

(11) $$N_{\mathrm{trap}}=\frac{2V_{\mathrm{TFL}}\varepsilon_{0}\varepsilon }{eL^{2}}$$

(12) $$J_{D}=\frac{9\mu \varepsilon \varepsilon_{0}V_{d}}{8L^{2}}$$

where *V*_TFL_, *ε*_0_, *ε*, *V*_d_, *J*_D_, and *L* are the trap‐filed limit voltage, vacuum permittivity, the relative dielectric constant (*ε*_InAs CQDs_ = 6), the applied voltage, current density at *V*_d_, and the device thickness, respectively.

### Transient Absorption Measurements

TA measurements were performed using a Femtosecond Transient Absorption Microscope (ST015) at the Korea Basic Science Institute. To measure the TA spectra, the samples were prepared on amorphous SiO_2_ slide glasses. Both pump and probe pulses were generated by a Yb:KGW regenerative amplifier (PHAROS, Light Conversion). One portion of the amplifier was used to pump a non‐collinear optical parametric amplifier (ORPHEUS‐N, Light Conversion), generating a pump beam at 450 nm. The absorbance of InAs CQDs layers was considerably higher at short wavelengths than at longer wavelengths. Considering the structure of the TA samples, 450 nm was chosen as the most appropriate pump wavelength for dominant generation of excitons in the InAs CQDs layer. For TA spectrum measurements, the probe beam was spectrally dispersed in a monochromator and detected by an electron‐multiplying charge‐coupled device camera triggered at 250 Hz.

## Conflict of Interest

The authors declare no conflict of interest.

## Supporting information

Supporting Information

## Data Availability

The data that support the findings of this study are available from the corresponding author upon reasonable request.
