# Supplementary material for: Ultrasensitive Near‐Infrared InAs Colloidal Quantum Dot‐ZnON Hybrid Phototransistor Based on a Gradated Band Structure
Source: Adv Sci (Weinh). 2023 Apr 23;10(18):2207526. doi: 10.1002/advs.202207526 (PMC10288248; doi:10.1002/advs.202207526)
Supplement: Supplementary file 1 — Supporting Information [file ADVS-10-2207526-s001.pdf]

ADVANCED SCIENCE
Open Access

Supporting Information

for *Adv. Sci.*, DOI 10.1002/advs.202207526

Ultrasensitive Near-Infrared InAs Colloidal Quantum Dot-ZnON Hybrid Phototransistor Based on a Gradated Band Structure

Jong-Ho Kim, Byung Ku Jung, Su-Kyung Kim, Kwang-Ro Yun, Junhyuk Ahn, Seongkeun Oh, Min-Gyu Jeon, Tae-Ju Lee, Seongchan Kim, Nuri Oh, Soong Ju Oh and Tae-Yeon Seong**

Copyright WILEY-VCH Verlag GmbH & Co. KGaA, 69469 Weinheim, Germany, 2018

Supporting Information

Ultrasensitive near-infrared InAs colloidal quantum dot-ZnON hybrid phototransistor based on a gradated band structure

Jong-Ho Kim[1], Byung Ku Jung[1], Su-Kyung Kim[1], Kwang-Ro Yun[1], Junhyuk Ahn[1], SeongKeun Oh[1], Min-Gyu Jeon[1], Tae-Ju-Lee[1], Seongchan Kim[2], Nuri Oh[2], Soong Ju Oh[1b], Tae-Yeon Seong[1a]

[1] Department of Materials Science and Engineering, Korea University, Seoul 02841, Korea

[2]Division of Materials Science and Engineering, Hanyang University, Seoul 04763, Korea

[a]Corresponding author: tyseong@korea.ac.kr (T.-Y. Seong)

[b]Corresponding author: sjoh1982@korea.ac.kr (S. J. Oh)

Keywords: hybrid phototransistor, III-V colloidal quantum dot, zinc oxynitride, InAs quantum dot, near-infrared detection, ligand engineering, surface chemistry

Materials	A_1	τ_1 [ps]	A_2	τ_2 [ps]
ZnON/InAs-ME	0.266	323.69	0.201	1198.62
ZnON/InAs-ME/InAs-$InCl_3$	0.253	202.74	0.235	1683.69

Table S1. TA decay curve parameters for ZnON/InAs-ME and ZnON/InAs-ME/InAs-$InCl_3$.

Materials	μ_{sat} [cm^2/Vs]	V_{th} [V]	SS [V/dec]	$I_{on/off}$
ZnON	73.8	-4.4	0.211	1.7×10^6
ZnON/InAs-ME	108.3	-8.1	0.181	5.9×10^6
ZnON/InAs-ME/InAs-$InCl_3$	120.1	-7.3	0.226	7.4×10^6

Table S2. Saturation mobility (μ_{Sat}), V_{th}, subthreshold swing (SS), and on/off ratio ($I_{on/off}$) of ZnON-only, ZnON/InAs-ME, and ZnON/InAs-ME/InAs-$InCl_3$ at dark state

Materials	Responsivity [A/W]	Detectivity [Jones]	Maximum EQE [%]	Rise time [s]	Fall time [s]
IGZO/InAs-ME	3.76×10^{3}	1.68×10^{13}	5.15×10^{5}	4.13	93.66
ZnON/InAs-ME	3.82×10^{4}	5.02×10^{14}	5.23×10^{6}	2.90	3.44

Table S3. Responsivity, detectivity, maximum external quantum efficiency (EQE), transient rise time, and fall time of ZnON/InAs-ME, and ZnON/InAs-ME/InAs-$InCl_3$

Light power density [W/cm^2]	External quantum efficiency (EQE) at V_G = -7 V [%]	
	ZnON/InAs-ME	ZnON/InAs-ME/InAs-$InCl_3$
2×10^{-6}	2.58×10^5	3.72×10^6
2×10^{-5}	1.92×10^5	9.74×10^5
2×10^{-4}	6.62×10^4	1.83×10^5
2×10^{-3}	1.30×10^4	2.14×10^4
2×10^{-2}	1.88×10^3	3.14×10^3

Table S4. Calculated EQE of ZnON/InAs-ME and ZnON/InAs-ME/InAs-$InCl_3$ under different light power densities at the gate voltage of -7 V.

Materials	Wavelength [nm]	Light intensity [W/cm^2]	Responsivity [A/W]	Detectivity [Jones]	Reference
IGZO/PbS QD	1300	1.19×10^{-8}	1.7×10^{3}	$10^{11} – 10^{12}$	[S1]
IGZO/PbS QD	980	1.2×10^{-6}	2.5×10^{2}	$10^{10} – 10^{11}$	[S2]
IGZO/PbS QD	1310	1.0×10^{-3}	$\sim 1.0 \times 10^{3}$	1.3×10^{12}	[S3]
Si/PbSe QD	1550	1.0×10^{-2}	6.1×10^{2}	4.8×10^{10}	[S4]
MoS_2/PbS QD	980	1.0×10^{-5}	5.4×10^{4}	1.0×10^{11}	[S5]
P3HT/HgTe QD	1550	2.2×10^{-6}	1.2×10^{1}	1.2×10^{10}	[S6]
WSe_2/PbS QD	970	2.5×10^{-5}	2.0×10^{5}	7.0×10^{13}	[S7]
PCBM/PbSe QD	1064	4.0×10^{-4}	2.8×10^{1}	1.3×10^{13}	[S8]
In_2O_3/BTPV4F:PTB7-Th	900	1.6×10^{-6}	1.4×10^{3}	4.8×10^{12}	[S9]
In_2O_3/PTPBT-ET	810	2.0×10^{-4}	2.0×10^{2}	1.2×10^{13}	[S10]
IZO/Organic BHJ	940	2.0×10^{-8}	1.8×10^{2}	5.0×10^{12}	[S11]
IGZO/DPP2ODT2-T (Tol)	850	1.0×10^{-5}	8.8×10^{3}	1.0×10^{13}	[S12]
DPPDTT:DCV3T	850	4.4×10^{-6}	2.0×10^{3}	1.0×10^{16}	[S13]
$MoS_2/MAPbI_3$	850	6.0×10^{-3}	1.0×10^{2}	2.4×10^{10}	[S14]
IGZO/$CsPb_xSn_{1-x}I$	860	7.8×10^{-3}	2.1×10^{1}	3.9×10^{10}	[S15]
MoS_2/Te	980	1.2×10^{-2}	2.8×10^{1}	2.7×10^{10}	[S16]
IGTO/TeO_x	970	1.0×10^{-4}	3.1×10^{1}	6.0×10^{11}	[S17]
$MoSe_2$-O_2	850	2.0×10^{-3}	5.6×10^{-1}	2.1×10^{13}	[S18]
Graphene/PbS QD	1280	$\sim 1.0 \times 10^{-4}$	7.2×10^{3}	8.4×10^{9}	[S19]
Graphene/Si QD	1450	2.0×10^{-7}	2.2×10^{9}	1.5×10^{13}	[S20]
ZnON/InAs-ME/InAs-$InCl_3$	905	2.0×10^{-6}	1.15×10^{5}	5.32×10^{16}	This work

Table S5. Responsivities and detectivities of NIR photodetectors previously investigated by other groups, which are displayed in Figure 6.

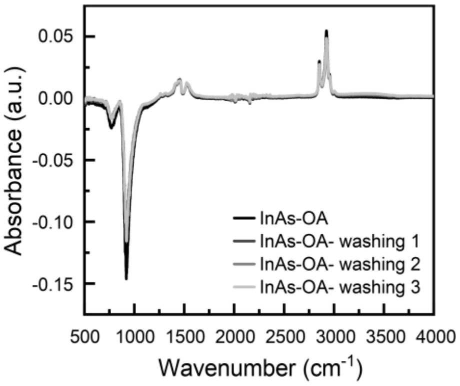

Figure S1. FTIR spectrum of InAs-OA before pure ethanol treatment, after one-, two-, and three-times treatment.

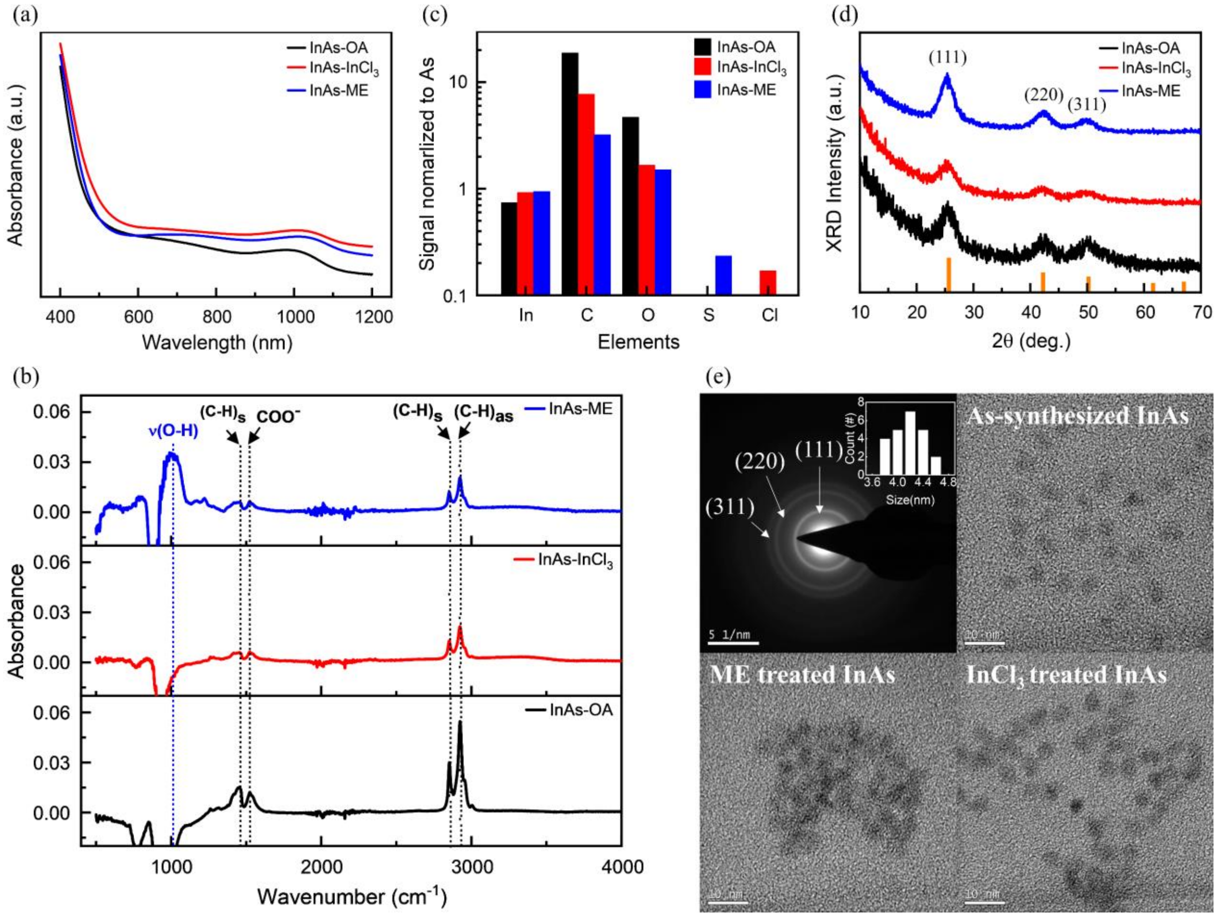

Figure S2. (a) UV-vis, (b) FT-IR, (c) XPS element analysis, and (d) XRD spectra of (black) as-synthesized InAs CQD (InAs-OA), (red) $InCl_3$-treated InAs CQD (InAs-$InCl_3$), and (blue) ME-treated InAs CQD (InAs-ME). (e) An SAED pattern and TEM images of InAs-OA (inset: corresponding histogram of the particle size distribution of CQDs). TEM images of InAs-$InCl_3$ and InAs-ME after the ligand exchange process.

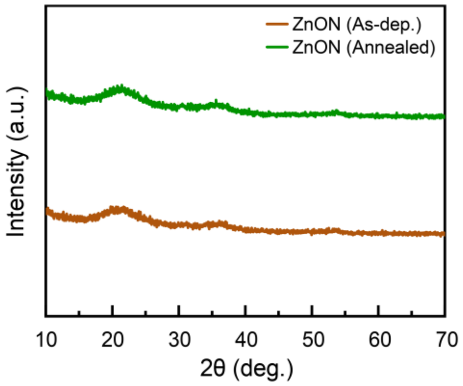

Figure S3. GIXRD spectra of ZnON films before and after post-annealing.

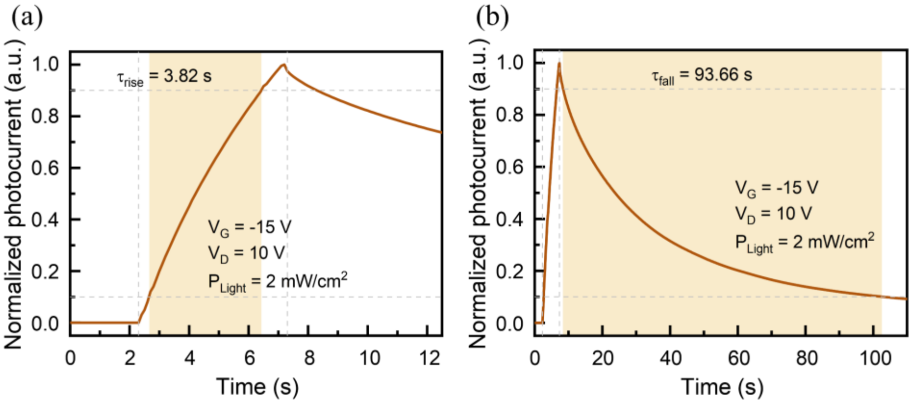

Figure S4. Transient photoresponse (a) rise and (b) fall times of IGZO/InAs-ME.

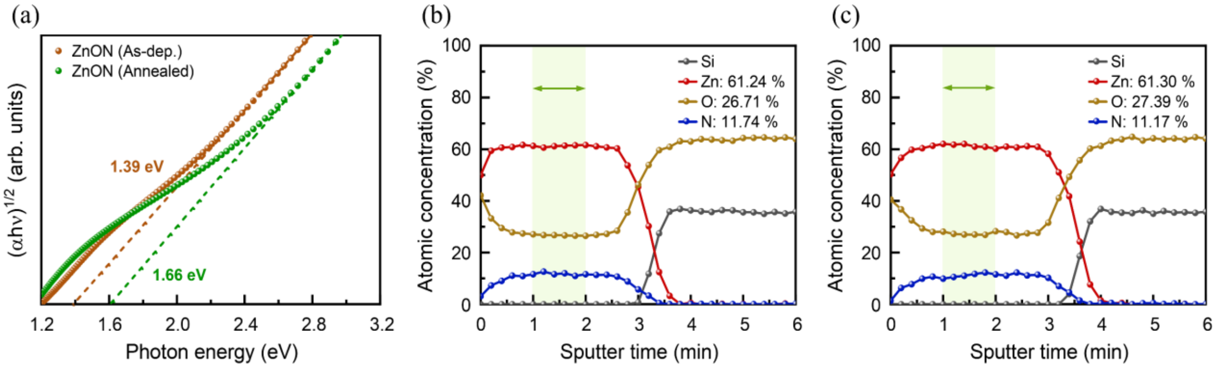

Figure S5. (a) Tauc plot of ZnON before and after annealing. Auger electron spectroscopy depth profile of ZnON film (b) before and (c) after post-annealing

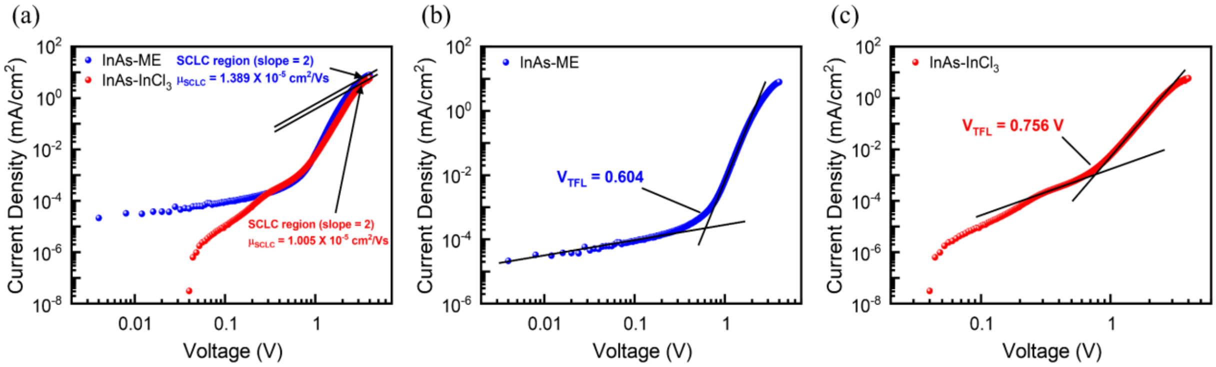

Figure S6. (a) Current-voltage curve of electron-only device (ITO/ZnO/InAs/ZnO/Au) using InAs-ME and InAs-$InCl_3$. Electron mobility of the two films calculated from the SCLC region (slope = 2) of the J-V curve using the Mott Gurney Law; InAs-ME (1.389×10^{-5} cm^2/Vs) and InAs-$InCl_3$ (1.005×10^{-5} cm^2/Vs), respectively. (b, c) The trap density of two films measured using the rapid current injection voltage (V_{TFL}) of the SCLC curve; InAs-ME (1.00×10^{16} cm^{-3}) and InAs-$InCl_3$ (1.25×10^{16} cm^{-3}).

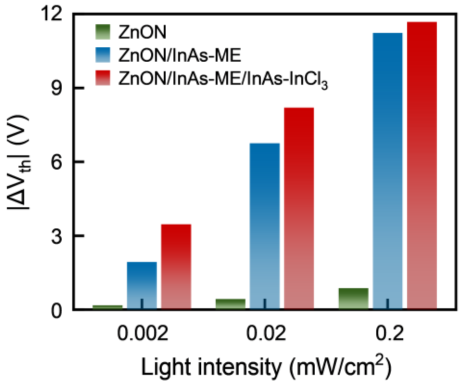

Figure S7. Threshold voltage (ΔV_{th}) changes of ZnON-only, ZnON/InAs-ME, and ZnON/InAs-ME/InAs-InCl$_3$ under 0.002 mW/cm^2, 0.02 mW/cm^2, and 0.2 mW/cm^2.

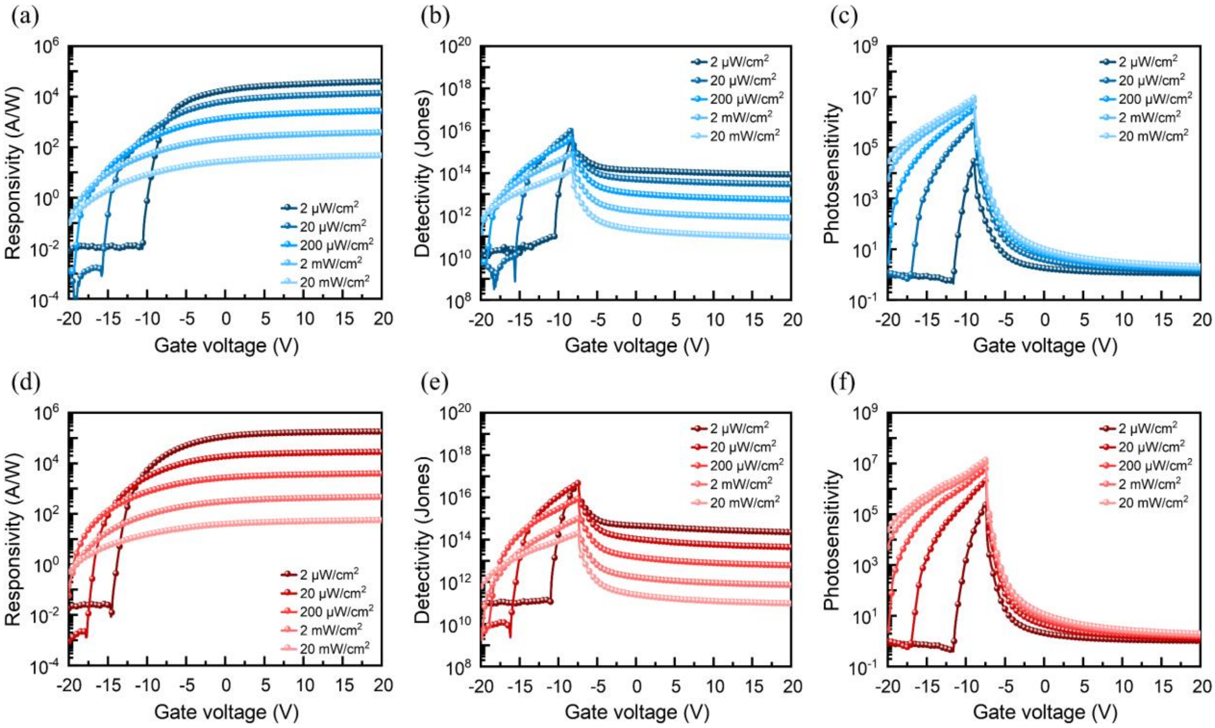

Figure S8. Gate voltage vs (a,d) responsivity, (b,e) detectivity, and (c,f) photosensitivity of (blue) ZnON/InAs-ME and (red) ZnON/InAs-ME/InAs-$InCl_3$ under 0.002 mW/cm^2, 0.02 mW/cm^2, 0.2 mW/cm^2, 2 mW/cm^2, and 20 mW/cm^2.

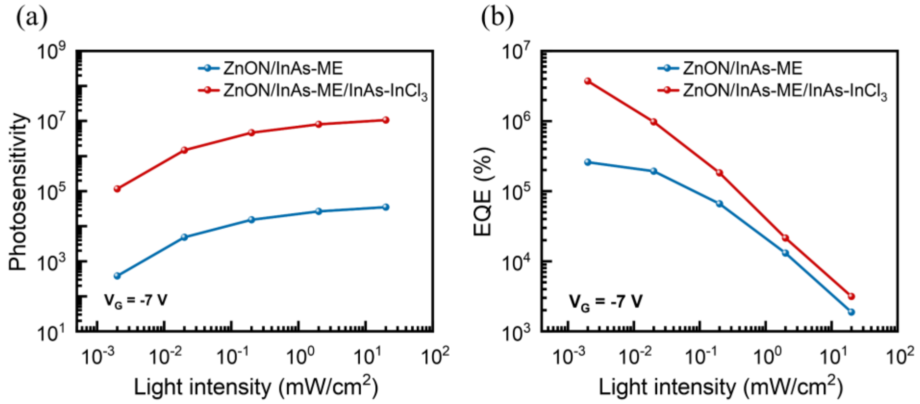

Figure S9. (a) Light dependency of photosensitivity was replotted from Figure S8c and S8f. The photosensitivity value at -7 V (gate voltage) of (red) ZnON/InAs-ME and (blue) ZnON/InAs-ME/InAs-$InCl_3$. (b) Light intensity-dependent external quantum efficiency (EQE) under different optical illumination power densities at the gate voltage of -7 V.

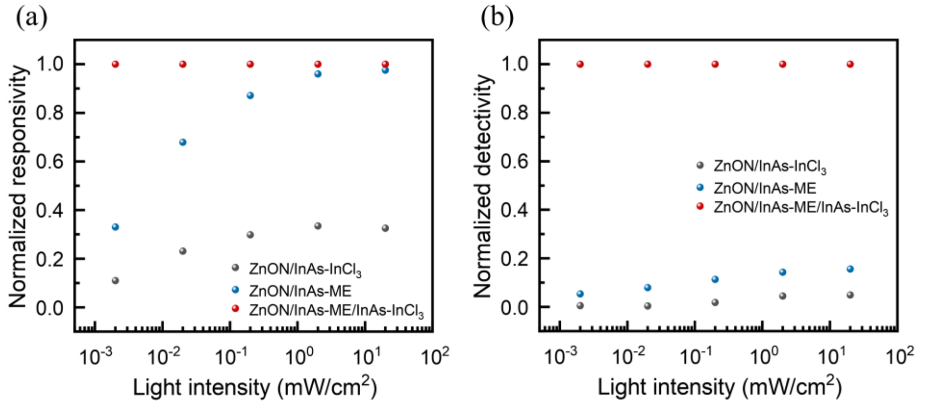

Figure S10. Normalized (a) responsivity and (b) detectivity of ZnON/InAs QD phototransistors. Normalization was performed by setting the performances of ZnON/InAs-ME/InAs-$InCl_3$ phototransistor to 1.

We fabricated a ZnON/InAs-$InCl_3$ device and compared its performance under different light intensity illuminated conditions. Under all conditions, the ZnON/InAs-$InCl_3$ exhibits lower responsivity and detectivity than ZnON/InAs-ME. The lower performance of the ZnON/InAs-$InCl_3$ is ascribed to relatively poorer ligand exchanges of $InCl_3$ than ME ligand.

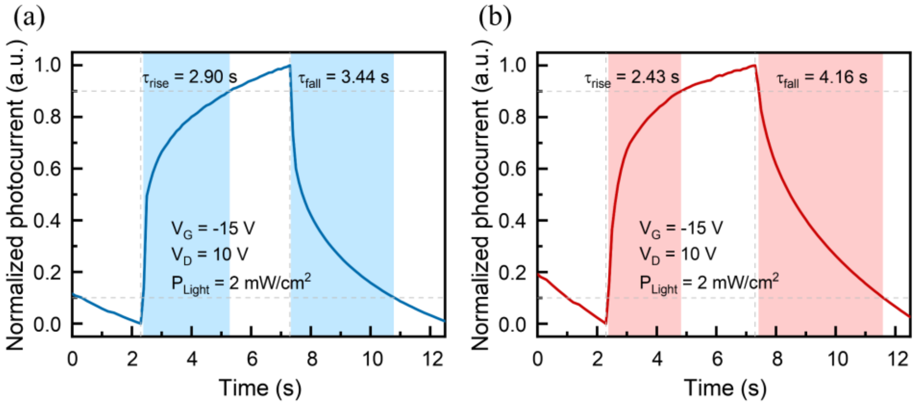

Figure S11. Transient photoresponse of (a) ZnON/InAs-ME and (b) ZnON/InAs-ME/InAs-$InCl_3$.

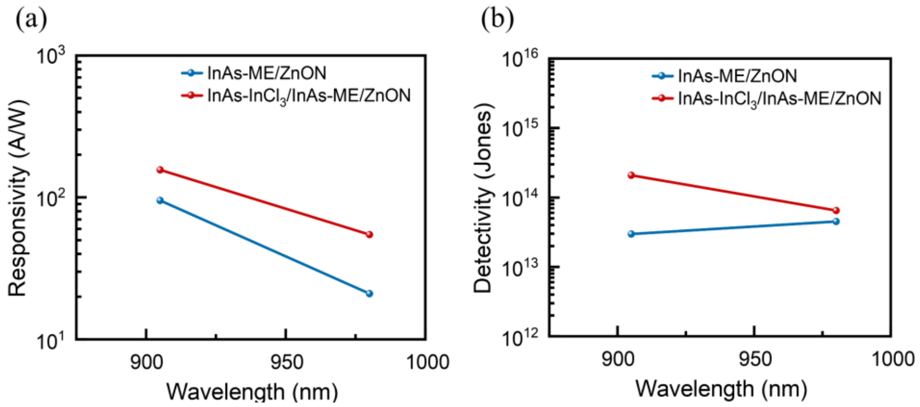

Figure S12. (a) Responsivities and (b) detectivities of ZnON/InAs-ME (905 nm and 980 nm) at the gate voltage of -7 V and the light power density of 2 mW/cm^2.

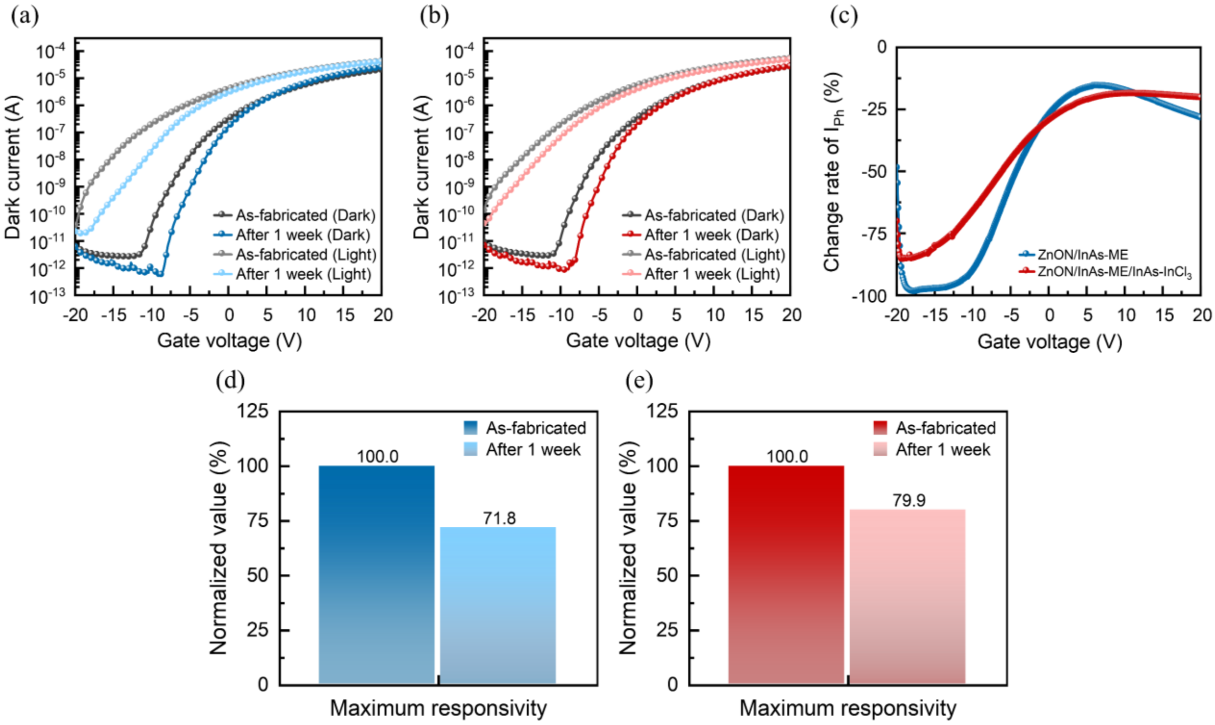

Figure S13. Transfer characteristics at the dark state and illuminated state of (a) ZnON/InAs-ME and (b) ZnON/InAs-ME/InAs-$InCl_3$ phototransistors immediately after fabrication and after leaving them in air for a week. The change rate of (c) I_{Ph} of ZnON/InAs-ME and ZnON/InAs-ME/InAs-$InCl_3$ phototransistors as a function of gate voltage. Comparison of the maximum responsivity of (d) ZnON/InAs-ME and (e) ZnON/InAs-ME/InAs-$InCl_3$ phototransistors before and after aging for 1 week in air. Performance of all phototransistors was measured at the light power density of 2 mW/cm^2.

Supporting Information Note 1

To measure the persistent photoconductivity (PPC) effect of ZnON, we integrated two different MOTP channels of ZnON and IGZO with InAs-ME. ZnON/InAs-ME and IGZO/InAs-ME were fabricated as described in the method section. Figure S4 and S11a show the transient photoresponse rise and fall times of IGZO/InAs-ME and ZnON/InAs-ME, respectively. ZnON/InAs-ME shows greatly faster photoresponse than IGZO/InAs-ME, where the rise time is 3.82 s and fall time is 93.66 s.

Supporting Information Note 2

As shown in Figure S4a, Figure S4b, and Figure S11a, compared to ZnON, IGZO shows much poor fall time due to the severe PPC effect. Many researchers reported that the PPC effect in IGZO is associated with the ionization of oxygen vacancy (V_O) and slow recovery, as given below,

$$V_O \text{ ionization: } V_O + 2h^+ \rightarrow V_O^{2+}$$

$$\text{Recovery: } V_O^{2+} + 2e^- \rightarrow V_O$$

When the light is turned off, the recovery of V_O^{2+} states is retarded by the energy barrier that hinders the neutralization of ionized V_O states. [S21]

In addition, another explanation for the cause of the PPC effect in IGZO is as follows. When IGZO is exposed to light, metastable peroxide states are formed within the band, acting as electron donors, as shown in the equation below. The recovery process is thermodynamically favorable. However, an energy barrier slows down the recovery, resulting in PPC effect.[S22]

$$\text{Peroxide formation: } 2O^{2-} + 2h^+ + 2e^- \rightarrow O_2^{2-} + 2e^-$$

$$\text{Recovery: } O_2^{2-} + 2e^- \rightarrow 2O^{2-}$$

Meanwhile, ZnON has an inactivated V_O state due to screening of N 2p orbitals constituting the valence band, and has fewer valence band tail states than IGZO. [S22] Therefore, the PPC effect is negligible in ZnON because both the ionization of V_O state and peroxide formation do not affect ZnON.[S26]

Supporting Information Note 3

In Table S3, ZnON shows significantly improved performance compared to IGZO. This performance enhancement is mainly attributed to the increase in mobility. High mobility promotes the recirculation of electrons in the channel until photogenerated holes decay, resulting in high photogain (G). Photogain is closely related to the responsivity (R): [S23-S25]

$$R \propto G$$

As shown in Table S2, the mobility of ZnON TFT is 73.8 cm^2/Vs, which is five times higher than that of IGZO TFT (14.1 cm^2/Vs). Responsivity is 10-fold increased by replacing IGZO by ZnON. This result implies that the high mobility of ZnON is an important factor contributing to the high performance of our phototransistors.

Supporting Information Note 4

In Figure S5b and S5c, AES depth profiles of the ZnON films before and after annealing at 250 °C for 1 hour in air were shown. The change rate (*CR*) of atomic concentration (*AC*) of Zn, O, and N in the ZnON films before and after annealing were calculated using following equation.

$$CR = \frac{(AC_{annealed\ ZnON} - AC_{as-deposited\ ZnON})}{AC_{as-deposited\ ZnON}} \times 100(\%)$$

in which $AC_{annealed\ ZnON}$ is atomic concentration of each element in the annealed ZnON film and $AC_{as\text{-}deposited\ ZnON}$ is atomic concentration of each element in the as-deposited ZnON film. Zn shows negligible change both in the surface (sputtering time of 0 ~ 0.4 min) and bulk (sputtering time of 1 ~ 2 min) of the ZnON film. However, other elements meaningfully changed after post-annealing in air. For example, the concentration of O is increased by 4.7% and N is decreased by 20.31% at the surface of ZnON after annealing. Thus, the nitrogen-deficient region formed by annealing causes the bandgap of ZnON to increase.

In addition, the estimated energy gap of 1.66 eV is larger than the energy of 905 nm light (1.37 eV), and this causes ZnON to hardly respond to NIR illumination. However, as the nitrogen-related defect states, such as nitrogen vacancy (V_N), in ZnON respond to light and ionization occurs as below,

$$V_N \rightarrow V_N^{3+} + 3e^-$$

$$V_N \rightarrow V_N^{+} + e^-$$

and form shallow donor levels close to the conduction band. [ref. 3-5] Therefore, the V_{th} shift of the ZnON-only device occurs at high light power density.

Supporting Information Note 5

As the band offset between the InAs-ME and ZnON layers becomes larger due to the increase of the ZnON bandgap, the hole movement from the InAs layer to the ZnON layer becomes more difficult than without annealing. Moreover, although not illustrated in this paper, the N-deficient ZnON region may make photoexcited electrons easily move to the carrier transport layer and at the same time interfere hole transfer.[S30]

References

[S1] D. K. Hwang, Y. T. Lee, H. S. Lee, Y. J. Lee, S. H. Shokouh, J.-H. Kyhm, J. Lee, H. H. Kim, T.-H. Yoo, S. H. Nam, D. I. Son, B.-K. Ju, M.-C. Park, J. D. Song, W. K. Choi, S. Im, *NPG Asia Mater.* **2016**, 8, e233.

[S2] H. T. Choi, J.-H. Kang, J. Ahn, J. Jin, J. Kim, S. Park, Y.-H. Kim, H. Kim, J. D. Song, G. W. Hwang, S. Im, W. Shim, Y. T. Lee, M.-C. Park, D. K. Hwang, *ACS Photonics* **2020**, 7, 1932.

[S3] J. Kim , S.-M. Kwon , Y. K. Kang , Y.-H. Kim , M.-J. Lee , K. Han , A. Facchetti, M.-G. Kim, S. K. Park, *Sci. Adv.* **2019**, 5, eaax8801.

[S4] Y. Shi, Z. Wu, X. Dong, P. Chen, J. Wang, J. Yang, Z. Xiang, M. Shen, Y. Zhuang, J. Gou, J. Wang, Y. Jiang, *Nanoscale* **2021**, 13, 12306.

[S5] S. Pak, Y. Cho, J. Hong, J. Lee, S. Lee, B. Hou, G.-H. An, Y.-W. Lee, J. E. Jang, H. Im, S. M. Morris, J. I. Sohn, S. Cha, J. M. Kim, *ACS Appl. Mater. Interfaces* **2018**, 10, 38264.

[S6] Y. Dong, M. Chen, W. K. Yiu, Q. Zhu, G. Zhou, S. V. Kershaw, N. Ke, C. P. Wong, A. L. Rogach, N. Zhao, *Adv. Sci.* **2020**, 7, 2000068.

[S7] C. Hu, D. Dong, X. Yang, K. Qiao, D. Yang, H. Deng, S. Yuan, J. Khan, Y. Lan, H. Song, J. Tang, *Adv. Funct. Mater.* **2017**, 27, 1603605.

[S8] A. Subramanian, S. Hussain, N. Din, G. Abbas, A. Shuja, W. Lei, J. Chen, Q. Khan, K. Musselman, *ACS Appl. Electron. Mater.* **2020**, 2, 3871.

[S9] D. Li, Z. Jia, Y. Tang, C. Song, K. Liang, H. Ren, F. Li, Y. Chen, Y. Wang, X. Lu, L. Meng, B. Zhu, *Nano Lett.* **2022**, 22, 5434.

[S10] D. Li, J. Du, Y. Tang, K. Liang, Y. Wang, H. Ren, R. Wang, L. Meng, B. Zhu, Y. Li, *Adv. Funct. Mater.* **2021**, 31, 2105887.

[S11] H. Kim, Z. Wu, N. Eedugurala, J. D. Azoulay, T. N. Ng, *ACS Appl. Mater. Interfaces* **2019**, 11, 36880.

[S12] B. H. Kang, K. Park, M. Hambsch, S. Hong, H. T. Kim, D. H. Choi, J. H. Lee, S. kim, H. J. Kim, *Nano Energy* **2022**, 92, 106773.

[S13] X. Jiang, J. Lu, D. Xue, Y. Wei, Y. Zhang, J. Zhang, Z. Wang, L. Huang, L. Chi, *Chem. Commun.* **2021**, 57, 12123.

[S14] D.-H. Kang, S. R. Pae, J. Shim, G. Yoo, J. Jeon, J. W. Leem, J. S. Yu, S. Lee, B. Shin, J.-H. Park, *Adv. Mater.* **2016**, 28, 7799.

[S15] C. Jo, S. Lee, J. Kim, J. S. Heo, D.-W. Kang, S. K. Park, *ACS Appl. Mater. Interfaces* **2020**, 12, 58038.

[S16] J. Yao, F. Chen, J. Li, J. Du, D. Wu, Y. Tian, C. Zhang, J. Yang, X. Li, P. Lin, *J. Mater. Chem. C* **2021**, 9, 13123.

[S17] H. Xu, T. Kim, H. Han, M. J. Kim, J. S. Hur, C. H. Choi, J.-H. Chang, J. K. Jeong, *ACS Appl. Mater. Interfaces* **2022**, 14, 3008.

[S18] Y. Huang, X. Zhou, L. Luo, J. Zou, H. Liu, X. Li, A. Ren, K. Shen, J. Wu, *Adv. Opt. Mater.* **2022**, 2200539.

[S19] M. J. Grotevent, C. U. Hail, S. Yakunin, D. Bachmann, G. Kara, D. N. Dirin, M. Calame, D. Poulikakos, M. V. Kovalenko, I. Shorubalko, *ACS Appl. Mater. Interfaces* **2021**, 13, 848.

[S20] Z. Ni, L. Ma, S. Du, Y. Xu, M. Yuan, H. Fang, Z. Wang, M. Xu, D. Li, J. Yang, W. Hu, X. Pi, D. Yang, *ACS Nano* **2017**, 11, 9854.

[S21] S. Jeon, S.-E. Ahn, I. Song, C. J. Kim, U.-I. Chung, E. Lee, I. Yoo, A. Nathan, S. Lee, K. Ghaffarzadeh, J. Robertson, K. Kim, *Nat. Mater.* **2012**, 11, 301.

[S22] J. T. Jang, J. Park, B. D. Ahn, D. M. Kim, S.-J. Choi, H.-S. Kim, D. H. Kim, *ACS Appl. Mater. Interfaces* **2015**, 7, 15570.

[S23] W. Zhang, C.-P. Chuu, J.-K. Huang, C.-H. Chen, M.-L. Tsai, Y.-H. Chang, C.-T. Liang, Y.-Z. Chen, Y.-L. Chueh, J.-H. He, M.-Y. Chou, L.-J. Li, *Sci. Rep.* **2015**, 4.

[S24] K. Chen, C. Zhang, X. Zang, F. Ma, Y. Chen, Y. Dan, *Small* **2021**, 17, 2006307.

[S25] A. De Iacovo, C. Venettacci, L. Colace, L. Scopa, S. Foglia, *Sci. Rep.* **2016**, 6, 37913.

[S26] J. T. Jang, H.-D. Kim, D. M. Kim, S.-J. Choi, H.-S. Kim, D. H. Kim, *IEEE Electron Device Lett.* **2020**, 41, 1376.

[S27] H.-M. Lee, H.-J. Jeong, K.-C. Ok, Y. S. Rim, J.-S. Park, *ACS Appl. Mater. Interfaces* **2018**, 10, 30541.

[S28] J. T. Jang, H.-D. Kim, C. Kim, S.-J. Choi, J.-H. Bae, D. M. Kim, H.-S. Kim, D. H. Kim, *IEEE Electron Device Lett.* **2021**, 42, 1006.

[S29] Y. S. Rim, K.-C. Ok, Y. M. Yang, H. Chen, S.-H. Bae, C. Wang, Y. Huang, J.-S. Park, Y. Yang, *ACS Appl. Mater. Interfaces* **2016**, 8, 14665.
